# Genetic Interactions with Sex Make a Relatively Small Contribution to the Heritability of Complex Traits in Mice

**DOI:** 10.1371/journal.pone.0096450

**Published:** 2014-05-08

**Authors:** Jon Krohn, Doug Speed, Rupert Palme, Chadi Touma, Richard Mott, Jonathan Flint

**Affiliations:** 1 Wellcome Trust Centre for Human Genetics, Oxford, United Kingdom; 2 UCL Genetics Institute, University College London, London, United Kingdom; 3 Department of Biomedical Sciences/Medical Biochemistry, University of Veterinary Medicine, Vienna, Austria; 4 Max Planck Institute of Psychiatry, Munich, Germany; Adamovic Research, Sweden

## Abstract

The extent to which sex-specific genetic effects contribute to phenotypic variation is largely unknown. We applied a novel Bayesian method, sparse partitioning, to detect gene by sex (GxS) and gene by gene (GxG) quantitative loci (QTLs) in 1,900 outbred heterogeneous stock mice. In an analysis of 55 phenotypes, we detected 16 GxS and 6 GxG QTLs. The increase in the amount of phenotypic variance explained by models including GxS was small, ranging from 0.14% to 4.30%. We conclude that GxS rarely make a large overall contribution to the heritability of phenotypes, however there are cases where these will be individually important.

## Introduction

Genome-wide association studies (GWAS) typically seek only main effects of genetic variation on phenotypes. While this methodology has succeeded in identifying quantitative trait loci (QTL), there are two reasons for being interested in interaction of genetics with other factors, such as sex and aspects of the organisms' environment. First, some loci might not be detected without taking interactions into account. Lander and colleagues have recently argued that a significant portion of ‘missing heritability’ in human GWAS is not due to the failure to detect sequence variants that contribute to phenotypic variation, but is hidden within unacknowledged interactions [1]. Second, identifying QTL involved in interactions might be important for understanding specific mechanisms, such as the biology of sex differences. It is possible that sex effects are manifest in a subset of the main effect QTL, but it is also possible that they represent a completely different set of loci whose biological function is restricted to the sex specific features of the phenotype.

Gene-by-sex (GxS) interaction QTL are genetic contributions to phenotypic variation that manifest themselves differently depending on the organism's sex. In contrast to sex as a main effect, which may induce sex-based dimorphism via broadly acting mechanisms like sex hormones, GxS interactions are associated with a specific locus of the genome and can account for phenotypic variance left unaccounted for by main effects alone. Observations from several species suggest that sex-specific genetic architecture plays a key role in the sex-based dimorphism of many traits, in Drosophila [2], mice [3], and rats [4], and quantitative traits associated with heart disease, hypertension, diabetes, asthma and autoimmune disease in humans [5].

Using crosses between inbred mouse lines, GxS interaction QTL have previously been identified for a number of phenotypes in mice, including body weight [6], [7], fat deposition [8], autoimmunity [9], and susceptibility to cancer [10], [11]. However, the poor mapping resolution inherent in designs that use inbred lines, and the relatively small number of phenotypes examined, leaves open the question of the extent to which GxS QTL contribute to phenotypic variation. Specifically, it is not clear to what extent GxS and main effect loci coincide, nor whether the contribution of GxS varies among phenotypes.

We set out to answer these questions using 55 phenotypes mapped at high resolution in heterogeneous stock (HS) mice. The HS mice are descended from eight inbred progenitors (A/J, AKR/J, BALB/cJ, C3H/HeJ, C57BL/6J, CBA/J, DBA/2J and LP/J [12]), each HS animal consisting of a fine-grained mosaic of the founder chromosomes, hence providing mapping of quantitative traits to an average resolution of about 3 Mb [13].

Mapping GxS loci in the HS has to deal with two problems. First, the degree of relatedness varies between individual HS mice so that mapping is more complicated than in classical inbred strain crosses; mapping in an HS has to take into account this population structure. Second, power to detect interactions is limited by the need to search through many possible combinations of predictors.

Both problems involve finding appropriate models, which is difficult to do with frequentist methods because of the large number of parameters that need to be fitted. Bayesian methods can be designed to deal with this situation by starting with more parameters than can be included in a frequentist approach. In this paper we used a Bayesian analytical tool called Sparse Partitioning [14] to map genetic loci and their interactions. Sparse Partitioning allows for models in which multiple predictors and their interactions influence outcome. This enables us to consider the contributions of GxS and also gene-by-gene interactions (GxG), or epistasis, on the phenotypes in the HS.

## Materials and Methods

### Ethics statement

Animal work was conducted according to the provisions of the UK Home Office. The protocol was approved by the Local Ethical Review Committee, Oxford University, and by a UK Home Office Project license. The protocol conforms to the principles of refinement, reduction and replacement and all tests were designed to minimize suffering.

### Animals and phenotypes

For this experiment we selected 55 phenotypes from fourteen different tests used to assess 1,900 HS mice. Data for this experiment are available from http://mus.well.ox.ac.uk/mouse/HS/and http://mus.well.ox.ac.uk/gscandb/. We selected these phenotypes from a larger set of 100, excluding those highly correlated within a test (for example, in the elevated plus maze, the time mice spent in the open arms was highly correlated with the number of times they entered the open arms), those with asymmetric highly skewed long-tailed distributions, and categorical and latency phenotypes. For an individual phenotype, *n* ranged from 712 to 1873, with a mean of 51.9% male (range of 50.6% to 56.0%; Table S2). Phenotypes that were highly non-Gaussian were normalised prior to analysis following the Box-Cox power transformation technique (Table S2). Phenotypes were adjusted for relatedness following the method described in [15].

### Fitting phenotype predictors

Our predictor set consisted of 12545 SNPs from across the whole genome, plus sex (any other known covariates, such as weight, if significant, were regressed out of phenotypes prior to analysis). To increase computational efficiency, wherever there were SNPs on the same chromosome with 99% concordance, only one of these SNPs was used for analysis (5332 SNPs remained). We applied a Bayesian method (Sparse Partitioning, or SP) that is designed to detect both main effects and interactions simultaneously [14]. SP defines models according to which predictors are associated with the phenotype and which of these predictors interact. SP was configured to settle upon models containing up to ten of the predictors defined in the above paragraph, with at most 2 three-way interactions between these predictors. SP models were iteratively fit over 2000 iterations of Markov Chain Monte Carlo. The first 500 iterations were discarded and the final 1500 used to calculate posterior probabilities. Thus, the posterior probability of association for each predictor is a fraction of 1500 representing the proportion of models in which that predictor was included. The posterior probability of two predictors interacting equals the proportion of models in which those two predictors are included and interact. For our primary SP analysis, any two predictors could interact (allowing both GxS and GxG interactions); to specifically assess the contribution of GxS interactions, we performed a secondary analysis in which only SNPs were allowed to interact (GxG). To obtain a false discovery rate at a given posterior probability threshold, we re-ran SP once for each phenotype using permuted response values. As shown in Figure S1, three interaction effects surpass the 0.2 posterior probability threshold we selected, suggesting we should expect a total of three false positives across all 55 phenotypes.

### Comparative fitting with resample model averaging

To confirm SP is appropriate for use with structured populations like the HS mice, we employed a resample model averaging method, Bagphenotype, to map main effect QTL for the same 55 phenotypes. Bagphenotype is an established tool for carrying out GWAS in HS animals and we ran it following a methodology described previously [13]. While SP is a Bayesian method that iteratively removes predictor variables from the models it fits, Bagphenotype is a frequentist method that iteratively adds predictors to the models it fits. With Bagphenotype, we ran one hundred bootstrapped multiple QTL regression models, resulting in a statistic called a resample model inclusion probability (RMIP) out of one hundred representing the strength of each predictive peak across the genome. A peak's RMIP represents the proportion of the multiple QTL models in which the peak's addition to the model both (1) improves the model fit, and (2) does not increase the model's adjusted *p*-value above a .05 significance threshold. Thus, a particular QTL that is included 70 times out of 100 based on these criteria, that QTL would be assigned an RMIP of .70. The genotypic data fed into Bagphenotype were founder haplotype probabilities, as estimated by HAPPY [16], for each interval between the 12545 genome-wide mouse SNPs. For a comparison with the GxS QTL identified by SP, Bagphenotype was also run using data from each sex alone.

### Heritability estimation

We estimated heritability, *h^2^*, by constructing from the SNP data a kinship matrix based on alleleic correlations [17], [18], then performing mixed model analysis supposing that the variation for each phenotype can be divided into an additive genetic component (with correlation structure specified by the kinship matrix) and an environmental component (corresponding to an identity matrix). We estimated the genetic and environmental components using REML [19], then our *h^2^* estimate for each phenotype was then the proportion of phenotypic variance estimated to be genetic.

## Results

**Figure 1:**
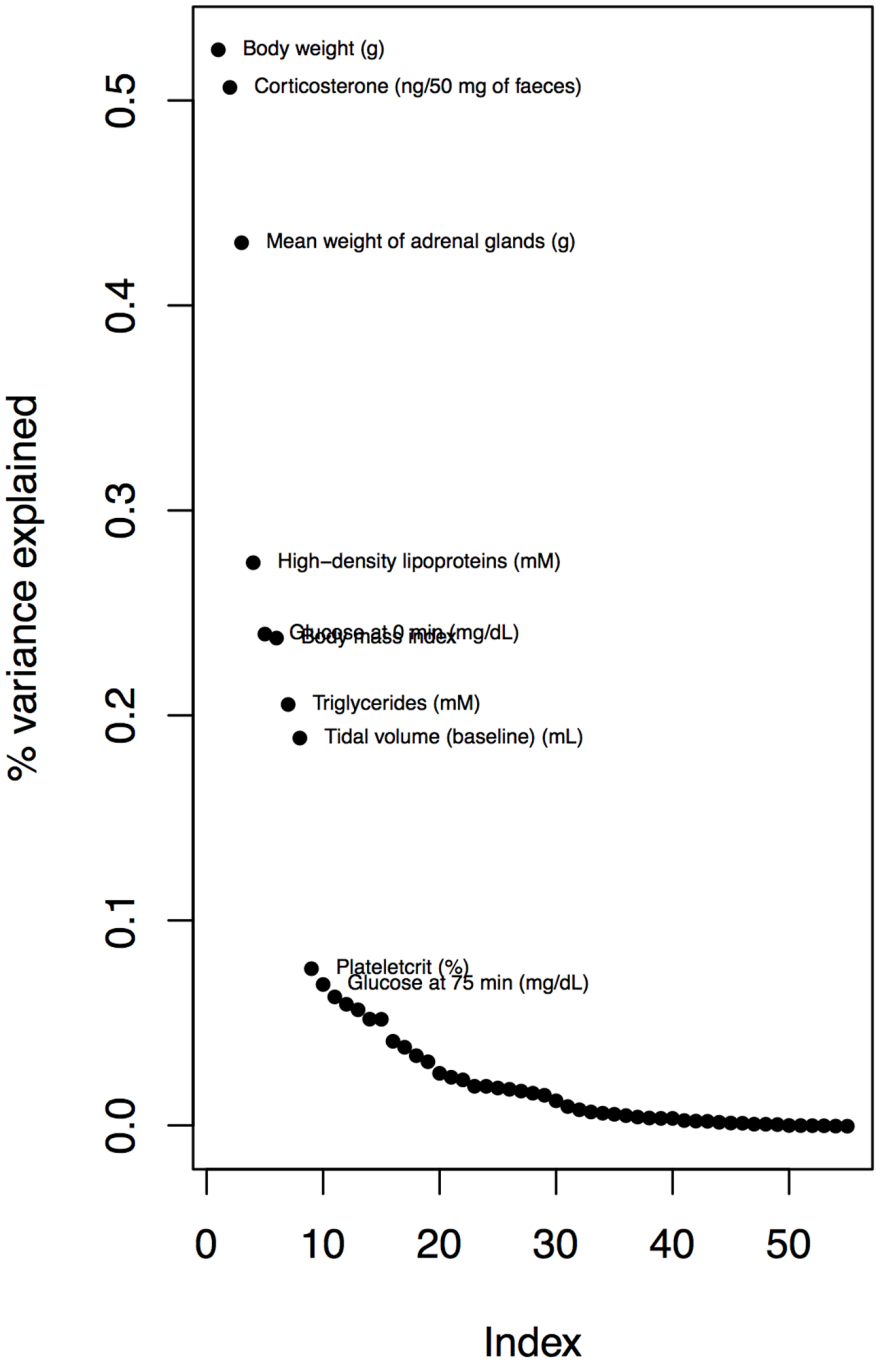
Main effects of sex on 55 heterogeneous stock mouse phenotypes. The vertical axis is the percent of variation explained. The ten largest effects are labeled.

**Figure 2:**
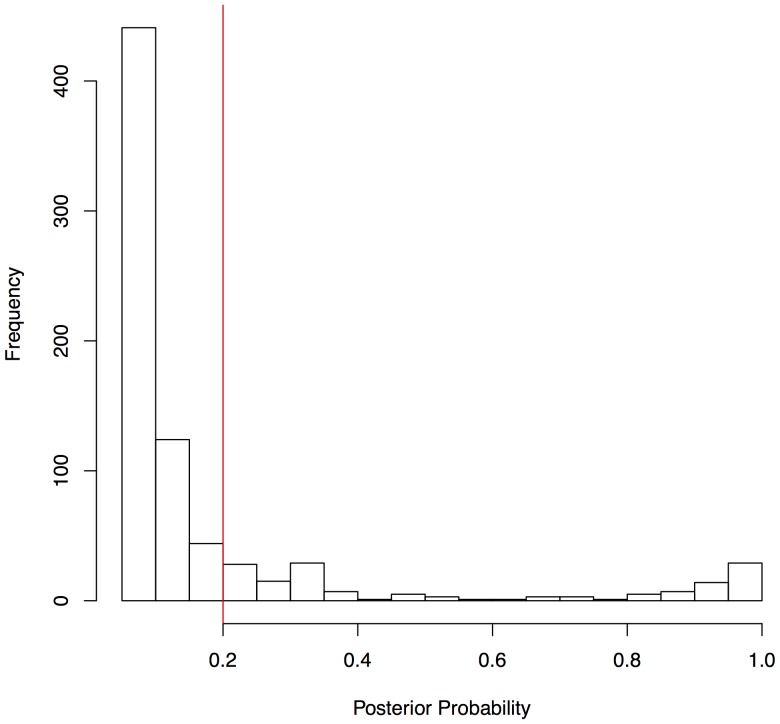
Histogram of the posterior probabilities of the main effect QTLs found by Sparse Partitioning. This histogram includes all main effect QTLs identified by Sparse Partitioning with a posterior probability greater than .05. The horizontal red line at .20 represents the threshold we selected: QTL above it were retained.

We employed SP to analyse 55 phenotypes in the HS and identified 47 that had a significant main effect of sex on the phenotype (at a 5% FDR). The distribution of effect sizes is highly skewed, ranging from 58% (body weight) to 0.2% (home cage activity), with a median of 2.4%. Figure 1 shows the distribution of effect sizes. Using SP, we identified 60 main effect loci from across the mouse genome (Table S1) above a posterior probability threshold of 0.2. As illustrated in Figure 2, the frequency of QTL below this threshold increases rapidly. QTL with posterior probabilities near zero are likely noise so these were discarded.

We compared SP main effect results with those from a resample model averaging approach (Bagphenotype) designed for use with structured populations like the HS mice [13]. Conservatively, we included only strictly overlapping QTL in our comparison of the two methods. The summary statistic for Bagphenotype is called a resample model inclusion probability (RMIP). For the 55 phenotypes, there are 294 QTL that exceed an RMIP >.25 threshold (an RMIP threshold of 0.25 was found by simulation to be equivalent to one false positive per genome wide scan [13]). Twenty-six (26/55 = 47.3%) of our SP-identified main effect QTL fell within 2 Mb of the 95% confidence interval (CI) of the Bagphenotype-identified main effect QTL.

We investigated whether there was greater consistency for QTL detected with higher certainty by each of the two methods. We found, as expected, that the posterior probabilities of the matched QTL (mean  = 0.64) were significantly higher than unmatched (mean  = 0.40), Wilcoxon rank sum test *W* = 581, *p*<.005. Similarly, the RMIPs of Bagphenotype QTL that matched (*n* = 26, mean  = 0.84) to SP QTL were significantly higher than those that did not (*n* = 268, mean  = .51; *W* = 6236, *p*<.0001).

**Figure 3:**
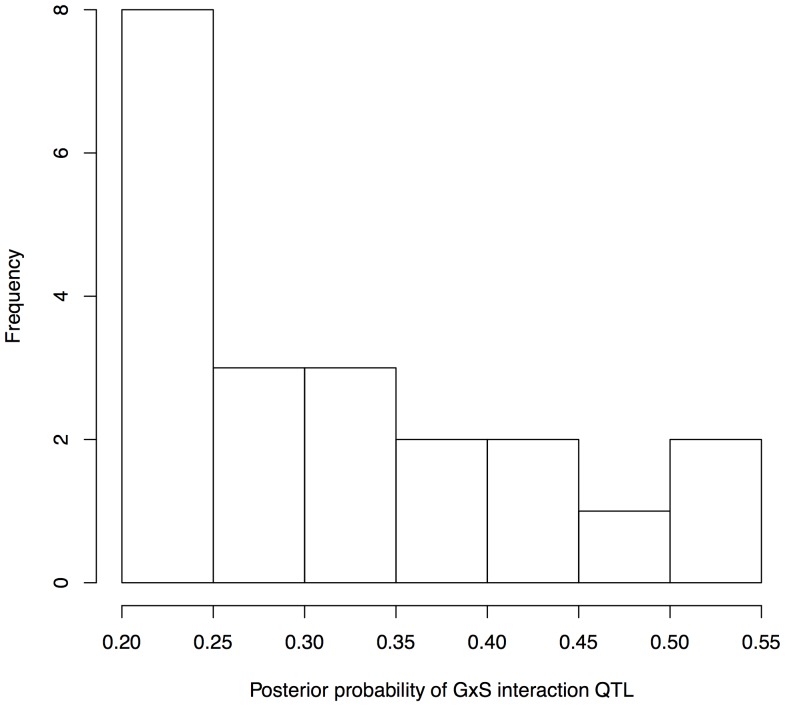
Histogram of the posterior probabilities of the GxS QTLs found by Sparse Partitioning. This histogram illustrates the frequency all GxS interaction QTL identified by SP with a posterior probability above the .20 threshold.

Setting the same posterior probability threshold for detecting interactions as main effects (0.20), SP detected 16 GxS interaction QTL (Table 1), summarized by a histogram of posterior probabilities in Figure 3, and well above the three false positives we expected at this threshold based on permutation analysis (Figure S1). These effects were associated with 15 of the 55 phenotypes investigated, with two GxS QTL for high-density lipoproteins (HDL) and one for each of the other 14 phenotypes. The highest GxS interaction posterior probability (.53) involved the time spent freezing in fearful context, and is located at 27.7 megabases (Mb) on chromosome 13.

**Table 1:** GxS QTL found by sparse partitioning.

Phenotype	SNP	Chr	Location (Mbp)	Posterior Probability	Minor Allele Frequency
Adrenal Gland Weight	rs3692711	7	20.63	0.29	0.245
Serum Alkaline Phosphatase	rs3719891	4	142.10	0.52	0.169
Serum Alanine Transaminase	CEL-X_113373391	X	50.44	0.32	0.201
Serum Chloride	rs13481037	11	55.61	0.20	0.221
Serum High-Density Lipoprotein	mCV24303778	4	115.92	0.43	0.472
Serum High-Density Lipoprotein	CEL-13_85845037	13	89.33	0.28	0.074
Serum Triglycerides	rs6299418	11	66.99	0.21	0.284
Freeze Time to Fear-Associated Context	CEL-13_27061395	13	27.77	0.53	0.247
Freeze Time to Fear-Associated Cue	rs3719988	6	73.68	0.20	0.246
Startle Response	CEL-11_120628029	11	120.82	0.35	0.376
Area Under Curve of Glucose Levels	mCV24984125	3	90.46	0.38	0.144
Hematocrit	rs3653651	11	102.01	0.37	0.211
CD4+ Cell% in CD3+ Cells	rs13481288	12	7.79	0.21	0.486
CD8+ Cell% in CD3+ Cells	rs3674782	16	87.32	0.24	0.179
B220+ Cell%	rs13475989	1	95.91	0.21	0.230
Boli Produced in Open Field Test	rs6163111	5	74.22	0.27	0.390

The GxS QTL identified by SP in many cases corresponded with main effect QTL we observed when using Bagphenotype on data from only one sex at a time. Of the 16 GxS QTL, six had a single-sex main effect QTL within 5 Mbp of the GxS QTL (female-only for adrenal gland weight and HDL cholesterol; male-only for alanine transaminase, triglycerides, startle response, and B220+ cell percentage) with no main effect QTL present nearby for the other sex. In only one instance, the alkaline phosphatase GxS QTL, was there both male- and female-only main effect QTL within 5 Mbp. Single-sex QTL scan results are provided in Table S3. This adds to the above finding of the considerable overlap (47.3%) of both-sexes-together main effects identified by SP and Bagphenotype. Although highly different mapping methods (SP's Bayesian, Bagphenotype's frequentist; SP removes model parameters, Bagphenotype adds them; SP uses three-level SNP allele data, Bagphenotype uses eight-level founder probabilities), we nevertheless observe consistency in both genetic main effect and GxS interaction QTL.

In addition to fitting GxS interactions, SP was configured to allow GxG interactions between QTL to explain variance in a phenotype. Seven such GxG interactions were identified above our 0.20 posterior probability threshold. Table 2 shows that each of the seven interactions is associated with a different phenotype; two also had GxS interactions (triglycerides and area of the glucose response curve) and four that did not (CD8+ T-cell count; blood glucose level; mean corpuscular hemoglobin, MCH; and mean corpuscular volume, MCV). The GxG interactions with the highest posterior probabilities (.45) were associated with MCH.

**Table 2:** GxG loci detected by sparse partitioning.

	QTL 1				QTL 2				
Phenotype	SNP	Chr	Location (Mbp)	MAF	SNP	Chr	Location (Mbp)	MAF	Posterior Probability
Serum Triglycerides	rs4213015	16	86.16	0.334	rs6249251	18	81.92	0.263	0.38
CD4+ Cell Count	rs13482751	15	102.78	0.367	mCV22965443	17	35.28	0.449	0.45
Body Weight	mCV23586427	1	191.63	0.334	rs13476524	2	58.51	0.013	0.27
AUC of Glucose Levels	mCV24984125	3	90.46	0.144	gnf12.073.387	12	76.40	0.324	0.31
Glucose Level After 0 Min.	rs3657255	1	35.09	0.306	rs3090050	11	95.21	0.430	0.31
Mean Corpuscular Haemoglobin	rs13482242	14	71.18	0.210	rs13482258	14	76.82	0.229	0.45
Mean Cellular Volume	rs6360170	14	69.28	0.429	rs13482239	14	70.17	0.364	0.32

To estimate how much phenotypic variance was attributable to GxS interactions, cross-validation was employed. Cross-validation randomly selects a proportion of the data with which the model is trained, and subsequently tests how well this model fits the unselected data. Here, we divided the data into ten evenly-sized tranches. Nine tranches were used to fit predictors to the data and then the quality of this fit was tested upon the tenth tranche. This was repeated ten times, allowing us to rotate through the ten tranches as the test tranche. We performed cross-validation twice with each of the 15 phenotypes that included a GxS interaction: first while allowing sex to act as both a marginal effect and as an interacting term with SNPs, then again while allowing sex to act only as a marginal effect. In both models, SNPs were able to interact (i.e., epistasis). The proportion of variance explained under these two scenarios is summarised in Table 3. The specific contribution of GxS interactions was assessed by considering how much more variance was explained under the scenario allowing GxS interactions.

**Table 3:** The percentage of phenotypic variance explained by two models: one permitting main and GxS effects, and one permitting only main effects.

Phenotype	Main and Interaction Effects Permitted	Only Main Effects Permitted	Difference
Adrenal Gland Weight	13.88	12.31	1.57
Serum Alkaline Phosphatase	6.09	5.88	0.21
Serum Alanine Transaminase	0.86	0.70	0.16
Serum Chloride	0.84	0.67	0.17
Serum High-Density Lipoprotein	9.37	8.83	0.54
Serum Triglycerides	3.31	3.02	0.29
Freeze Time to Fear-Associated Context	−0.05	−0.37	0.32
Freeze Time to Fear-Associated Cue	6.34	6.24	0.10
Startle Response	5.26	5.05	0.21
AUC of Glucose Levels	0.50	0.35	0.14
Hematocrit	0.14	−0.39	0.53
CD4+ Cell% in CD3+ Cells	3.05	2.82	0.23
CD8+ Cell% in CD3+ Cells	0.40	−0.13	0.54
B220+ Cell%	0.81	0.40	0.41
Boli Produced in Open Field Test	−0.15	−0.48	0.33

The best fitting models are for adrenal weight, where the GxS interaction-prohibited and -permitted models explain 12.3% and 13.9% of the phenotypic variance, respectively. The proportion of variance explained by the GxS interaction-permitted models was higher than in their GxS interaction-prohibited counterparts, ranging from 0.14% (area of the glucose response curve) to 4.30% (body weight) higher. Other than adrenal weight (1.57%), allowing GxS interactions did not account for more than about half a percent of the phenotypic variance.

**Figure 4:**
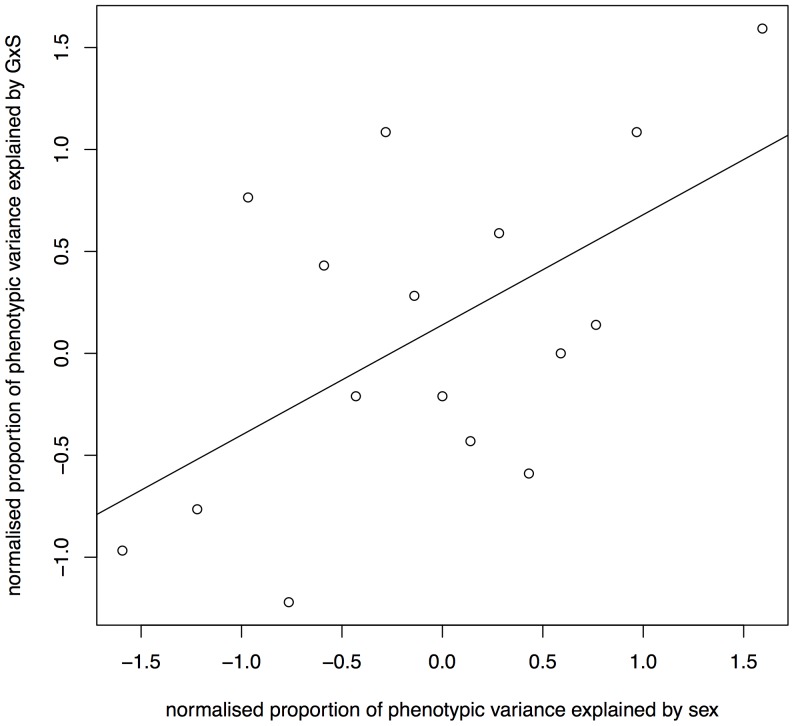
Correlation between GxS and main effect of sex. The proportion of phenotypic variance explained by sex as a main effect is plotted on the horizontal axis and the proportion of variance explained by the interaction effect is shown on the vertical axis. The two correlate with a P value <0.05.

Across the 15 phenotypes with a GxS QTL identified by SP, the proportion of phenotypic variance explained by sex as a main effect (determined by a simple linear model) correlates with the proportion of variance explained by GxS interactions, *r* = .57, *t*(14)  = 2.6, *p*<.05 (column four, “difference”, in Table 3). The correlation is shown in Figure 4.

Heritability estimates, *h^2^*, for all phenotypes are provided in Table S4. *h^2^* did not correlate significantly with the proportion of phenotypic variance explained by main effects alone, nor by the variance explained by main and interaction effects together, nor with the proportion of variance explained by GxS interactions (columns two through four in Table 3; *p*>.1 in all three cases).

## Discussion

Our principle finding is that GxS interactions contribute little to phenotypic variation in addition to that attributable to main effect QTL. While we found GxS QTL at just over a quarter of phenotypes, the median percentage of variation accounted for by a GxS QTL was 0.23% (Table 3). By contrast, the main effect QTL had a median contribution of 6.2% (maximum  = 24.7%). Furthermore, since every GxS QTL we identified coincides with one of the 60 main effect QTL (Table S1), our data indicate that the GxS effect does not arise from biological pathways independent of those of the main effect QTL.

The search for GxG interactions yielded an even smaller list of loci. Seven epistatic interactions were found, each for a different phenotype (Table 2). Three of the GxG interactions coincided precisely with main effect QTL (loci interacting to predict triglyceride levels, loci for area under the curve of glucose levels, loci for CD4+ cell count). Two others (for mean corpuscular haemoglobin (MCH) and mean cellular volume) lie within 2 Mb of main effect QTL.

Attempts to find sex specific effects have met with varying success, partly because of methodological limitations. A review of the literature on human genetic association studies, despite identifying 432 claims for sex-specificity, concluded that the majority of claims were spurious [20]. Yet there is evidence from twin and genetic linkage studies that, for some phenotypes, a considerable proportion of the genetic variance is sex specific. Thus in two independent twin studies of the heritability of depression, Kendler and colleagues [21], [22] estimate that genetic correlation in risk factors for major depression in men and women to be approximately 0.6. In an analysis of 17 quantitative phenotypes, subject to genetic linkage analysis, Ober and colleagues reported that eleven were sexually dimorphic, twelve showed evidence of differences between the sexes in heritability or linkage, and all three genome-wide significant linkage peaks were significant when tested for an interaction between sex and genotype [5].

Our results add to this debate by finding evidence for GxS at just over a quarter of phenotypes, suggesting that GxS QTL are relatively common, in agreement with the genetic linkage analyses of human phenotypes [5]. Furthermore, we observed a linear relationship between sex effects and GxS: the larger the main effect of sex, the larger the effect of the interaction loci (Figure 4). This justifies the reasonable assumption that it will be worth examining highly sexually dimorphic phenotypes for GxS effects. This linear relationship between sex effects and GxS might be interpreted to mean that the genetic basis of sex differences arise from the conjoint effect of many loci, rather than being due to a specific and relatively constrained biological pathway. However it is important to realize that the distribution of effect sizes we observed is skewed. This may indicate that in some phenotypes the sex effect arises from a few key loci. One example might be the GxS interaction based at 21 Mbp on chromosome seven that explains 1.5% of adrenal gland weight.

One important caveat to our approach is that we found relatively few main effect loci. Compared to the 294 main effect QTL found with a model averaging mapping method, SP found just 60 loci, of which 43% were common to the two methods. This raises the question as to whether reliance on SP for QTL mapping might be biasing our results. It is surprising, for example, that we found no GxS for weight, even though this phenotype has by far the largest sex effect of any phenotype we measured (58%, Figure 1). This is most likely due to our relatively low power, given the expected small effects of each GxS QTL.

**Figure 5:**
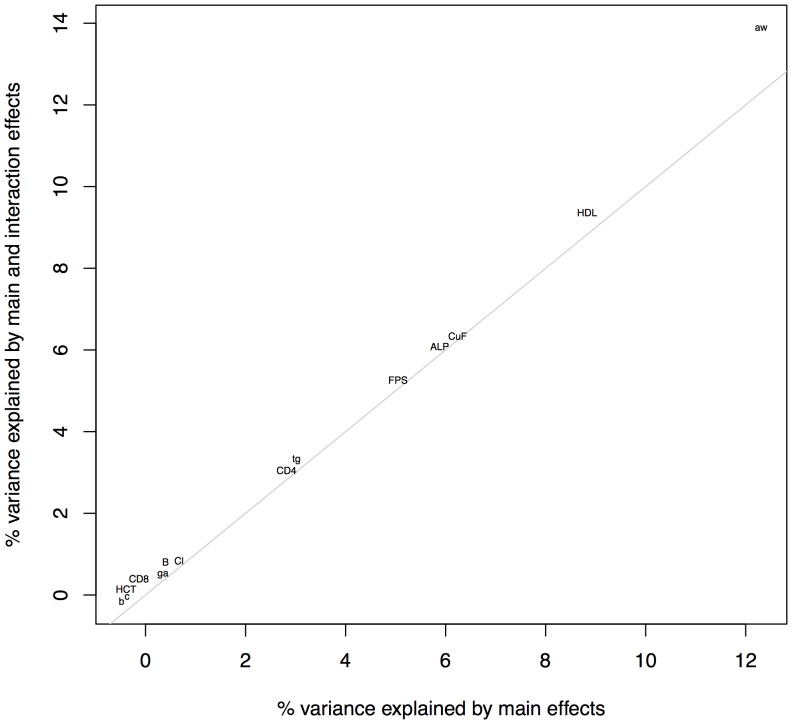
Proportion of phenotypic variance explained by GxS versus by main effects. For the 15 phenotypes that had a GxS QTL, this plot depicts the percentage of phenotypic variance explained by models where sex was permitted to act as a main effect only relative to when it could act as a both main effect and interaction term. As detailed in Table 3, allowing GxS interaction effects in the model at least marginally improved the amount of phenotypic variance explained by predictors. Thus, all the points fall above the grey line, x = y. It is clear from the figure that adrenal gland weight (“aw”) had the greatest improvement in its variance explained by allowing interacting predictors (difference of 1.6%). Additional non-trivial abbreviations are as follows: chloride (“Cl”), triglycerides (“tg”), time spent frozen in fearful context (“c”), time spent frozen after fearful cue (“CuF”), startle response (“FPS”), area of glucose response curve (“ga”), B220+ cell percentage (“B”), and boli produced in the open field test (“b”). ALT occupied nearly the same position as chloride so is represented by the same symbol (“Cl”).

While the smaller numbers of main effects found with SP suggests that more interacting loci might exist, it does not invalidate our main finding of the paucity of interacting loci and their small effect size, relative to main effect loci. For example reducing the posterior probability threshold for SP results would not identify sufficient additional GxS effects to alter our conclusion that this set of loci makes only a small contribution to the total genetic variance (Figure 5).

The advantage of using SP is that it is less subject to the problem of multiple testing. Like other sparse Bayesian methods, SP allows for very complicated models defined by a very large number of parameters. It does this by assuming most parameters are zero (i.e., that most predictors do not influence the outcome either marginally or through interactions). This type of approach is particularly effective in situations where the number of predictors in a model greatly exceeds the sample size (the large *p*, small *n* problem). For GxG interactions, where a genome scan would involve testing pairwise interactions between thousand of loci, identifying significant effects would otherwise require extremely large sample sizes, too large to be feasible.

Finally, although we found that first-order interactions contribute little to phenotypic variation beyond main effect QTL and other covariates, we emphasize that this does not imply that they can be ignored. One example is the GxS interaction based at 21 Mbp on chromosome seven influencing adrenal gland weight. While GxS rarely make a large overall contribution to the missing heritability of phenotypes, there are cases where there will be individually important.

## Supporting Information

Figure S1 **Posterior probabilities of interaction effects obtained using permuted values for each of the phenotypes.**
(DOCX)

Table S1 **Main effect QTL identified by sparse partitioning.**
(DOCX)

Table S2 **By phenotype, the Box-Cox power technique-identified transform to Gaussian distribution, the number of HS mice, and number of mice that were male.**
(DOCX)

Table S3 **Main effect QTL identified using resample model averaging and data from only one sex. Those within 5 Mbp of GxS QTL identified by sparse partitioning (** **Table 1** **) are shown in bold.**
(DOCX)

Table S4 **Heritability estimates across phenotypes.**
(DOCX)
