# Supplementary figures and images for: Genetic Interactions with Sex Make a Relatively Small Contribution to the Heritability of Complex Traits in Mice
Source: PLoS One. 2014 May 8;9(5):e96450. doi: 10.1371/journal.pone.0096450 (PMC4014490; doi:10.1371/journal.pone.0096450)

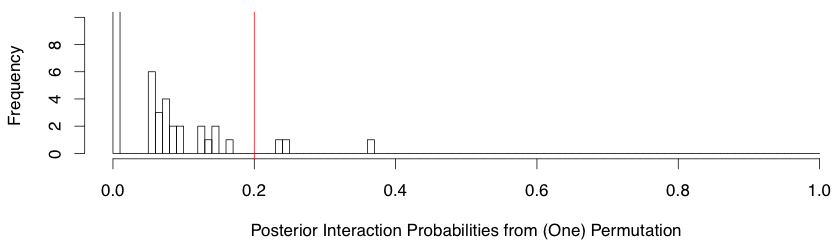

Supplement: Figure S1 — Posterior probabilities of interaction effects obtained using permuted values for each of the phenotypes. (DOCX) [file pone.0096450.s001.docx]
