# Supplementary material for: Genetic Interactions with Sex Make a Relatively Small Contribution to the Heritability of Complex Traits in Mice
Source: PLoS One. 2014 May 8;9(5):e96450. doi: 10.1371/journal.pone.0096450 (PMC4014490; doi:10.1371/journal.pone.0096450)
Supplement: Table S1 — Main effect QTL identified by sparse partitioning. (DOCX) [file pone.0096450.s002.docx]

| **Phenotype** | **SNP** | **Chr** | **Location (Mbp)** | **Posterior Probability** |
| --- | --- | --- | --- | --- |
| Adrenal Gland Weight | CEL-7_13089324 | 7 | 25.14 | 0.29 |
| Serum Alkaline Phosphatase | rs13478006 | 4 | 137.28 | 0.85 |
| Serum Alanine Transaminase | rs13483757 | X | 50.44 | 0.43 |
| Serum Chloride | rs13479859 | 8 | 84.57 | 0.25 |
| Serum Chloride | rs13481037 | 11 | 55.61 | 0.20 |
| Serum High-Density Lipoprotein | UT_1_175.440644 | 1 | 173.51 | 0.75 |
| Serum High-Density Lipoprotein | UT_4_114.441786 | 4 | 110.21 | 0.43 |
| Serum High-Density Lipoprotein | CEL-13_85845037 | 13 | 89.33 | 0.28 |
| Serum Phosphorous | rs3164088 | 16 | 96.54 | 0.30 |
| Serum Triglycerides | rs13481078 | 11 | 67.00 | 0.21 |
| Serum Triglycerides | rs4213015 | 16 | 86.16 | 0.37 |
| Serum Triglycerides | rs6249251 | 18 | 81.92 | 0.40 |
| Serum Urea | rs6283871 | 17 | 3.48 | 0.58 |
| CD4+ Cell Count | rs3694208 | 8 | 146.96 | 0.21 |
| CD8+ Cell Count | rs13482751 | 15 | 102.78 | 0.30 |
| CD8+ Cell Count | mCV22965443 | 17 | 35.28 | 0.63 |
| Freeze Time to Fear-Associated Context | CEL-13_27061395 | 13 | 27.78 | 0.53 |
| Freeze Time to Fear-Associated Cue | rs3719988 | 6 | 73.68 | 0.20 |
| Freeze Time to Fear-Associated Cue | rs6326790 | 15 | 90.38 | 1.00 |
| Ear Hole Area Six Weeks After Ear Punch | rs6213614 | 7 | 89.43 | 0.95 |
| Body Weight | mCV23386455 | 5 | 64.58 | 0.25 |
| Body Weight | rs13483825 | X | 71.45 | 0.94 |
| Body Weight | rs13484003 | X | 130.14 | 0.69 |
| Faecal Corticosterone Metabolites | rs3708913 | 7 | 25.94 | 0.24 |
| Startle Response | CEL-11_120628029 | 11 | 120.82 | 0.85 |
| Startle Response | rs6326790 | 15 | 90.38 | 1.00 |
| Area Under Curve of Glucose Levels | mCV24984125 | 3 | 90.46 | 0.76 |
| Area Under Curve of Glucose Levels | gnf12.073.387 | 12 | 76.40 | 0.30 |
| Glucose Levels After 75 Minutes | rs13477814 | 4 | 84.52 | 0.25 |
| Basophils | rs13479465 | 7 | 120.05 | 0.20 |
| Basophils | rs3089065 | 11 | 93.03 | 0.29 |
| Basophils | rs13481991 | 13 | 106.11 | 0.30 |
| Hematocrit | rs3653651 | 11 | 102.01 | 0.37 |
| Mean Corpuscular Haemoglobin | rs3724826 | 1 | 133.40 | 0.42 |
| Mean Corpuscular Haemoglobin | rs6248193 | 1 | 157.31 | 1.00 |
| Mean Corpuscular Haemoglobin | rs8249856 | 8 | 125.49 | 0.23 |
| Mean Corpuscular Haemoglobin | rs6366991 | 9 | 107.92 | 0.89 |
| Mean Corpuscular Haemoglobin | rs6393401 | 11 | 5.77 | 0.93 |
| Mean Corpuscular Haemoglobin | UT_14_62.125092 | 14 | 74.52 | 0.79 |
| Mean Cellular Volume | rs13476111 | 1 | 133.65 | 0.40 |
| Mean Cellular Volume | rs6248193 | 1 | 157.34 | 0.58 |
| Mean Cellular Volume | rs4222040 | 11 | 56.85 | 0.40 |
| Mean Platelet Volume | rs8237062 | 1 | 173.15 | 0.99 |
| Mean Platelet Volume | rs6357939 | 11 | 96.94 | 0.20 |
| White Blood Cell Count | CEL-X_94143306 | X | 99.93 | 0.44 |
| CD4+:CD8+ Cell Ratio | rs13482957 | 17 | 34.17 | 0.98 |
| CD4+ Cell Intensity | rs13481288 | 12 | 77.94 | 0.22 |
| CD4+ Cell Intensity | rs13481816 | 13 | 54.78 | 0.21 |
| CD4+ Cell Intensity | rs3686467 | 19 | 16.96 | 0.97 |
| CD8+ Cell Intensity | rs3674782 | 16 | 87.32 | 0.24 |
| B220+ Cell % | rs13475989 | 1 | 95.91 | 0.21 |
| CD3+ Cell % | rs13476242 | 1 | 175.30 | 0.87 |
| CD3+ Cell % | rs4223448 | 2 | 126.93 | 0.22 |
| CD4+ Cell % in CD3+ Cells | mCV22965443 | 17 | 35.28 | 0.97 |
| CD8+ Cell % in CD3+ Cells | mCV22965443 | 17 | 35.28 | 0.98 |
| Area Under Curve of IR Insulin Response | rs6309331 | 9 | 92.17 | 0.31 |
| KI-67 Antigen | rs13479023 | 6 | 127.39 | 0.33 |
| Body Mass Index | rs13459165 | 2 | 128.96 | 0.44 |
| Boli Produced in Open Field Test | rs6163111 | 5 | 74.22 | 0.27 |
| Plethysmography Tidal Volume Baseline | rs3089065 | 11 | 93.03 | 0.24 |
