# Supplementary material for: Genetic Interactions with Sex Make a Relatively Small Contribution to the Heritability of Complex Traits in Mice
Source: PLoS One. 2014 May 8;9(5):e96450. doi: 10.1371/journal.pone.0096450 (PMC4014490; doi:10.1371/journal.pone.0096450)
Supplement: Table S2 — By phenotype, the Box-Cox power technique-identified transform to Gaussian distribution, the number of HS mice, and number of mice that were male. (DOCX) [file pone.0096450.s003.docx]

| Experiment | Phenotype measure | Transform to Gaussian | *n_total_* | *n_male_* |
| --- | --- | --- | --- | --- |
| Activity in new home cage | Total beam breaks due to ambulation | x^1/2^ | 1905 | 981 |
| Adrenal gland weight | Mean weight of adrenal glands (g) | log_10_x | 1863 | 960 |
| Context freezing | Time freezing to context (sec) | x^1/2^ | 1374 | 723 |
| Cue freezing | Time freezing during cue (sec) | x | 1518 | 802 |
| Elevated plus maze | Closed-arm distance (cm) | x | 1850 | 960 |
|  | Open-arm distance (cm) | x^1/3^ | 1882 | 975 |
| Fear-potentiated startle | Startle response | x^1/3^ | 1642 | 851 |
| Faecal steroid | Corticosterone (ng/50 mg of faeces) | log_10_x | 1090 | 560 |
| KI-67 | KI-67 antigen | x | 715 | 381 |
| Open field test | Fecal boli count | x | 1925 | 993 |
| Animal size | Body weight (g) | x^1/3^ | 1873 | 959 |
|  | Body length (cm) | x | 1885 | 988 |
|  | Body mass index | x | 1869 | 973 |
| Biochemistry | Albumin (g/L) | x | 1735 | 895 |
|  | Alkaline phosphatase (units/L) | x^1/2^ | 1752 | 904 |
|  | Alanine transaminase (units/L) | log_10_(x + 3) | 1651 | 863 |
|  | Aspartate transaminase (units/L) | x^-1/2^ | 1690 | 886 |
|  | Calcium (mM) | x | 1740 | 894 |
|  | Chloride (mM) | x | 1791 | 921 |
|  | Creatinine (mg/dL) | rank of x | 1929 | 996 |
|  | High-density lipoproteins (mM) | x | 1653 | 850 |
|  | Low-density lipoproteins (mM) | log_10_x | 1701 | 877 |
|  | Phosphorous (mM) | log_10_(x + 1) | 1536 | 793 |
|  | Total protein (g/L) | x^2^ | 1629 | 824 |
|  | Triglycerides (mM) | log_10_x | 1503 | 841 |
|  | Urea (mM) | log_10_(x + 1) | 1732 | 888 |
| IPGTT | Area Under Curve – Glucose (mg/dL) | x | 1771 | 929 |
|  | Area Under Curve – Insulin (ng/mL) | x^1/2^ | 1379 | 708 |
|  | Glucose at 0 min (mg/dL) | x^1/2^ | 1849 | 952 |
|  | Glucose at 75 min (mg/dL) | x^1/2^ | 1784 | 935 |
| Ear punch | Mean area of hole in ear (mm^2^) | x^-1/2^ | 1823 | 942 |
| Full blood count | Basophil count | rank of x | 1929 | 996 |
|  | Hematocrit (%) | x^3^ | 1583 | 819 |
|  | Hemoglobin (g/dl) | x^3^ | 1580 | 816 |
|  | Lymphocytes (1000/mm^3^) | x^1/2^ | 1554 | 812 |
|  | Mean cellular hemoglobin (g/dL) | x | 1581 | 818 |
|  | Mean cellular volume (um^3^) | x | 1585 | 821 |
|  | Mean platelet volume (um^3^) | rank of x | 1929 | 996 |
|  | Monocyte count | rank of x | 1929 | 996 |
|  | Neutrophil count | rank of x | 1929 | 996 |
|  | Plateletcrit (%) | x^1/2^ | 1552 | 803 |
|  | Platelets (units/microL) | x | 1574 | 814 |
|  | RBC count (1000000/mm^3^) | x^3^ | 1580 | 817 |
|  | WBC count (1000/mm^3^) | log_10_(x + 1) | 1563 | 816 |
| Immunology | B220+ cells (%) | x^1/2^ | 1516 | 784 |
|  | CD3+ cells in lymphocytes (%) | x^2^ | 1515 | 782 |
|  | CD4+ cells in CD3+ cells (%) | x^2^ | 1514 | 784 |
|  | CD4+ cell intensity | x^2^ | 1513 | 784 |
|  | CD4+ count | x | 1269 | 673 |
|  | CD8+ cells in CD3+ cells (%) | x^-1/3^ | 1512 | 782 |
|  | CD8+ cell intensity | x | 1509 | 782 |
|  | CD8+ count | x | 1267 | 671 |
|  | CD4+:CD8+ ratio | x^-1/3^ | 1510 | 781 |
| Plethysmography | Respiratory rate (baseline) | x | 1812 | 950 |
|  | Tidal volume (baseline) (mL) | x^1/3^ | 1801 | 939 |
