# Supplementary material for: Genetic Interactions with Sex Make a Relatively Small Contribution to the Heritability of Complex Traits in Mice
Source: PLoS One. 2014 May 8;9(5):e96450. doi: 10.1371/journal.pone.0096450 (PMC4014490; doi:10.1371/journal.pone.0096450)
Supplement: Table S3 — Main effect QTL identified using resample model averaging and data from only one sex. Those within 5 Mbp of GxS QTL identified by sparse partitioning ( Table 1 ) are shown in bold. (DOCX) [file pone.0096450.s004.docx]

| Phenotype | Sex | Chr | Range1 (bp) | Range2 (bp) | RMIP |
| --- | --- | --- | --- | --- | --- |
| Adrenal Gland Weight | female | 2 | 121731696 | 124239391 | 0.31 |
| Adrenal Gland Weight | female | 2 | 138033487 | 145107318 | 0.26 |
| Adrenal Gland Weight | female | 4 | 122943300 | 125265532 | 0.45 |
| Adrenal Gland Weight | female | 6 | 34603223 | 38495238 | 0.5 |
| **Adrenal Gland Weight** | **female** | **7** | **24837074** | **27852765** | **0.32** |
| Adrenal Gland Weight | female | 7 | 111381390 | 114746266 | 0.28 |
| Adrenal Gland Weight | female | X | 129783295 | 133124932 | 0.79 |
| Adrenal Gland Weight | male | 1 | 130485574 | 135256658 | 0.31 |
| Adrenal Gland Weight | male | 3 | 29004632 | 39215658 | 0.3 |
| Adrenal Gland Weight | male | 6 | 38804725 | 40081576 | 0.31 |
| Adrenal Gland Weight | male | 7 | 3176074 | 6911682 | 0.28 |
| Serum Albumin | female | 1 | 73785852 | 75283346 | 0.35 |
| Serum Albumin | female | 6 | 111302621 | 114213243 | 0.38 |
| Serum Albumin | female | 11 | 32646121 | 34127561 | 0.95 |
| Serum Albumin | female | 19 | 10248920 | 16955850 | 0.54 |
| Serum Albumin | male | 4 | 25691755 | 27943470 | 0.25 |
| Serum Albumin | male | 18 | 68674028 | 70283257 | 0.78 |
| Serum Alkaline Phosphatase | female | 1 | 130668783 | 133564393 | 0.6 |
| **Serum Alkaline Phosphatase** | **female** | **4** | **134974246** | **139804324** | **1** |
| Serum Alkaline Phosphatase | female | 5 | 65434363 | 67053013 | 0.36 |
| Serum Alkaline Phosphatase | female | 9 | 103527697 | 107607630 | 0.37 |
| Serum Alkaline Phosphatase | female | 12 | 107058673 | 109140900 | 0.25 |
| Serum Alkaline Phosphatase | female | 15 | 28975560 | 30896669 | 0.26 |
| Serum Alkaline Phosphatase | female | 17 | 33517617 | 43780952 | 0.31 |
| **Serum Alkaline Phosphatase** | **male** | **4** | **135525051** | **141170941** | **1** |
| Serum Alkaline Phosphatase | male | 5 | 89422254 | 93062967 | 0.37 |
| Serum Alkaline Phosphatase | male | 6 | 43905925 | 47731981 | 0.52 |
| Serum Alkaline Phosphatase | male | 19 | 22628546 | 24783638 | 0.26 |
| Serum Alkaline Phosphatase | male | X | 106221447 | 115361139 | 0.77 |
| Serum Alanine Transaminase | female | 1 | 186627043 | 187552612 | 0.29 |
| Serum Alanine Transaminase | female | 2 | 161278733 | 165221683 | 0.88 |
| Serum Alanine Transaminase | female | X | 98027281 | 101301320 | 0.33 |
| Serum Alanine Transaminase | male | 2 | 150449200 | 153029515 | 0.37 |
| Serum Alanine Transaminase | male | 3 | 97507155 | 101413815 | 0.43 |
| Serum Alanine Transaminase | male | 4 | 127685633 | 130281463 | 0.31 |
| **Serum Alanine Transaminase** | **male** | **X** | **49663745** | **51078517** | **0.35** |
| Serum Aspartate Aminotransferase | female | 3 | 28979184 | 30845875 | 0.39 |
| Serum Aspartate Aminotransferase | female | 8 | 105860113 | 106368411 | 0.25 |
| Serum Aspartate Aminotransferase | female | 11 | 57574025 | 57644675 | 0.4 |
| Serum Aspartate Aminotransferase | female | 12 | 55550090 | 55988845 | 0.71 |
| Serum Aspartate Aminotransferase | male | 1 | 37372166 | 38555850 | 0.28 |
| Serum Aspartate Aminotransferase | male | 9 | 103604005 | 104015736 | 0.69 |
| Serum Aspartate Aminotransferase | male | 11 | 67705817 | 69031637 | 0.3 |
| Serum Calcium | female | 2 | 138305655 | 141777975 | 0.67 |
| Serum Calcium | female | 3 | 11119213 | 19033618 | 0.27 |
| Serum Calcium | female | 5 | 148799645 | 152187052 | 0.47 |
| Serum Calcium | female | 19 | 15843635 | 20275251 | 0.33 |
| Serum Calcium | male | 3 | 97507155 | 98035113 | 0.73 |
| Serum Calcium | male | 5 | 5431978 | 8916439 | 0.66 |
| Serum Calcium | male | 8 | 101142950 | 105277540 | 0.33 |
| Serum Calcium | male | 15 | 58447977 | 64422310 | 0.57 |
| Serum Calcium | male | 16 | 8838458 | 10700907 | 0.38 |
| Serum Chloride | female | 1 | 73464630 | 75283346 | 0.3 |
| Serum Chloride | female | 2 | 52430672 | 53042542 | 0.34 |
| Serum Chloride | female | 3 | 80665579 | 81299007 | 0.28 |
| Serum Chloride | female | 5 | 10670977 | 13998275 | 0.26 |
| Serum Chloride | female | 5 | 148792855 | 150849955 | 0.41 |
| Serum Chloride | female | 8 | 47724730 | 49778676 | 0.4 |
| Serum Chloride | female | 15 | 10505166 | 15646749 | 0.33 |
| Serum Chloride | female | 15 | 18084710 | 20105239 | 0.27 |
| Serum Chloride | female | 18 | 34792252 | 39107143 | 0.33 |
| Serum Chloride | female | 19 | 18712951 | 22263388 | 0.61 |
| Serum Chloride | male | 2 | 144810472 | 145857974 | 0.3 |
| Serum Chloride | male | 3 | 97507155 | 98763055 | 0.67 |
| Serum Chloride | male | 5 | 5431978 | 8916439 | 0.29 |
| Serum Chloride | male | 5 | 45254182 | 49876647 | 0.33 |
| Serum Chloride | male | 8 | 90852652 | 93011781 | 0.46 |
| Serum Chloride | male | 11 | 67962799 | 72150322 | 0.57 |
| Serum Chloride | male | 13 | 4597936 | 11898065 | 0.38 |
| Serum Chloride | male | 15 | 66752114 | 68411183 | 0.25 |
| Serum High-Density Lipoprotein | female | 1 | 173512974 | 175295409 | 1 |
| Serum High-Density Lipoprotein | female | 3 | 80665579 | 81299007 | 0.63 |
| **Serum High-Density Lipoprotein** | **female** | **4** | **108770805** | **111199155** | **0.62** |
| **Serum High-Density Lipoprotein** | **female** | **4** | **114037242** | **117083314** | **0.35** |
| Serum High-Density Lipoprotein | female | 7 | 31205452 | 31244528 | 0.43 |
| Serum High-Density Lipoprotein | female | 7 | 73843465 | 76700446 | 0.34 |
| Serum High-Density Lipoprotein | female | 8 | 48616114 | 53645706 | 0.34 |
| Serum High-Density Lipoprotein | female | 10 | 43355687 | 44882191 | 0.27 |
| Serum High-Density Lipoprotein | female | 10 | 68029824 | 70917635 | 0.44 |
| Serum High-Density Lipoprotein | female | 18 | 44316600 | 49028056 | 0.29 |
| Serum High-Density Lipoprotein | female | X | 153742601 | 156801190 | 0.57 |
| Serum High-Density Lipoprotein | male | 1 | 171235205 | 173631425 | 1 |
| Serum High-Density Lipoprotein | male | 4 | 103313211 | 110238889 | 0.68 |
| Serum High-Density Lipoprotein | male | 5 | 128047790 | 135611538 | 0.44 |
| Serum High-Density Lipoprotein | male | 6 | 45986359 | 51845790 | 0.27 |
| Serum High-Density Lipoprotein | male | 10 | 101837924 | 102461576 | 0.37 |
| Serum High-Density Lipoprotein | male | 15 | 62952529 | 63489848 | 0.32 |
| Serum High-Density Lipoprotein | male | 15 | 81657003 | 85458326 | 0.34 |
| Serum Low-Density Lipoprotein | female | 1 | 184271870 | 185401055 | 0.46 |
| Serum Low-Density Lipoprotein | female | 4 | 89292013 | 91009109 | 0.29 |
| Serum Low-Density Lipoprotein | female | 4 | 128200051 | 133435313 | 0.26 |
| Serum Low-Density Lipoprotein | female | 5 | 24760691 | 31562855 | 0.27 |
| Serum Low-Density Lipoprotein | female | 15 | 77135358 | 81657003 | 0.46 |
| Serum Low-Density Lipoprotein | female | 16 | 83406529 | 85165653 | 0.35 |
| Serum Low-Density Lipoprotein | female | 19 | 23295631 | 26429667 | 0.5 |
| Serum Low-Density Lipoprotein | male | 1 | 185385632 | 186627043 | 0.38 |
| Serum Low-Density Lipoprotein | male | 2 | 156698689 | 158683823 | 0.46 |
| Serum Low-Density Lipoprotein | male | 4 | 98811889 | 109541448 | 0.52 |
| Serum Low-Density Lipoprotein | male | 11 | 32327598 | 37271257 | 0.41 |
| Serum Phosphorous | female | 3 | 40783983 | 41907956 | 0.33 |
| Serum Phosphorous | female | 4 | 38269852 | 40396917 | 0.33 |
| Serum Phosphorous | female | 16 | 93538251 | 96039450 | 0.38 |
| Serum Phosphorous | female | 18 | 80279943 | 87142544 | 0.48 |
| Serum Phosphorous | male | 2 | 36091355 | 38382286 | 0.56 |
| Serum Phosphorous | male | 3 | 86709915 | 87781832 | 0.31 |
| Serum Phosphorous | male | 4 | 130913579 | 131555056 | 0.29 |
| Serum Phosphorous | male | 5 | 6240949 | 7869718 | 0.27 |
| Serum Phosphorous | male | 6 | 147672016 | 149016561 | 0.26 |
| Serum Phosphorous | male | 8 | 13824360 | 15993277 | 0.6 |
| Serum Phosphorous | male | 9 | 112348235 | 115055684 | 0.32 |
| Serum Phosphorous | male | 11 | 36127399 | 39837716 | 0.39 |
| Serum Phosphorous | male | 15 | 82049630 | 89873413 | 0.44 |
| Serum Total Protein | female | 8 | 117662831 | 120068815 | 0.32 |
| Serum Total Protein | male | 3 | 68376449 | 69819671 | 0.27 |
| Serum Total Protein | male | 6 | 23915122 | 24365592 | 0.29 |
| Serum Total Protein | male | 15 | 32283162 | 34447594 | 0.27 |
| Serum Total Protein | male | 16 | 27868813 | 29670024 | 0.67 |
| Serum Triglycerides | female | 3 | 78863357 | 81299007 | 0.52 |
| Serum Triglycerides | female | 7 | 31220697 | 38888026 | 0.48 |
| Serum Triglycerides | female | 7 | 76635293 | 77309744 | 0.34 |
| Serum Triglycerides | female | 7 | 132726486 | 134927348 | 0.28 |
| Serum Triglycerides | female | 10 | 111836341 | 113842314 | 0.41 |
| Serum Triglycerides | male | 3 | 58293740 | 61269680 | 0.57 |
| Serum Triglycerides | male | 4 | 155234947 | 155495757 | 0.27 |
| **Serum Triglycerides** | **male** | **11** | **66810493** | **69633779** | **0.66** |
| Serum Triglycerides | male | 13 | 54124947 | 55212325 | 0.7 |
| Serum Triglycerides | male | 16 | 11750190 | 12887956 | 0.66 |
| Serum Triglycerides | male | X | 90183557 | 95472354 | 0.35 |
| Serum Urea | female | 1 | 117626679 | 120636961 | 0.34 |
| Serum Urea | male | 2 | 66543092 | 67059205 | 0.36 |
| Body Weight | female | 3 | 140551055 | 142720742 | 0.5 |
| Body Weight | female | 5 | 20266652 | 22716575 | 0.42 |
| Body Weight | female | 5 | 136305277 | 138095206 | 0.55 |
| Body Weight | female | 13 | 88826415 | 93772727 | 0.32 |
| Body Weight | female | X | 129783295 | 132263611 | 0.27 |
| Body Weight | male | 5 | 57923864 | 65141892 | 0.4 |
| Body Weight | male | 6 | 19885952 | 22074735 | 0.39 |
| Body Weight | male | 10 | 36136190 | 36343122 | 0.44 |
| Body Weight | male | 19 | 3185700 | 3717162 | 0.48 |
| Body Weight | male | X | 148331809 | 150142966 | 0.26 |
| CD4+ Count | female | 5 | 46065650 | 49959139 | 0.26 |
| CD4+ Count | female | 7 | 111297435 | 114746266 | 0.5 |
| CD4+ Count | female | 9 | 13010473 | 15693572 | 0.37 |
| CD4+ Count | female | 10 | 20971210 | 21502038 | 0.25 |
| CD4+ Count | female | 18 | 74895730 | 75971158 | 0.47 |
| CD4+ Count | male | 5 | 13253142 | 14804960 | 0.29 |
| CD4+ Count | male | 5 | 28165863 | 29413722 | 0.27 |
| CD4+ Count | male | 5 | 109059308 | 111537717 | 0.34 |
| CD4+ Count | male | 8 | 14696094 | 15152117 | 0.44 |
| CD4+ Count | male | 8 | 23258929 | 24559322 | 0.45 |
| CD4+ Count | male | 9 | 59934477 | 65397435 | 0.46 |
| CD4+ Count | male | 13 | 52660661 | 54980347 | 0.26 |
| CD4+ Count | male | 15 | 68909584 | 75435371 | 0.27 |
| CD8+ Count | female | 16 | 54656264 | 56982171 | 0.67 |
| CD8+ Count | female | 18 | 16225937 | 18676154 | 0.68 |
| CD8+ Count | male | 5 | 16220992 | 16515714 | 0.38 |
| CD8+ Count | male | 8 | 14758999 | 15993277 | 0.52 |
| CD8+ Count | male | 14 | 63105448 | 64881411 | 0.33 |
| CD8+ Count | male | 16 | 88158178 | 94690274 | 0.51 |
| Freeze Time to Fear-Associated Context | female | 6 | 135332855 | 136560667 | 0.27 |
| Freeze Time to Fear-Associated Context | female | 13 | 114908768 | 118393689 | 0.28 |
| Freeze Time to Fear-Associated Context | female | 14 | 47011225 | 48876075 | 0.39 |
| Freeze Time to Fear-Associated Context | male | 4 | 26576934 | 30348906 | 0.31 |
| Freeze Time to Fear-Associated Context | male | 13 | 43266279 | 48107521 | 0.78 |
| Freeze Time to Fear-Associated Context | male | 17 | 11997072 | 13825489 | 0.47 |
| Freeze Time to Fear-Associated Context | male | 17 | 46723440 | 50872569 | 0.31 |
| Freeze Time to Fear-Associated Context | male | 19 | 30621558 | 32123399 | 0.43 |
| Freeze Time to Fear-Associated Cue | female | 11 | 55207955 | 56514996 | 0.26 |
| Freeze Time to Fear-Associated Cue | female | 13 | 41224351 | 48013737 | 0.34 |
| Freeze Time to Fear-Associated Cue | female | 15 | 88996113 | 91723248 | 1 |
| Freeze Time to Fear-Associated Cue | male | 15 | 91723248 | 92992022 | 0.99 |
| Ear Hole Area Six Weeks After Ear Punch | female | 1 | 159872834 | 163488843 | 0.36 |
| Ear Hole Area Six Weeks After Ear Punch | female | 1 | 186268950 | 187710901 | 0.29 |
| Ear Hole Area Six Weeks After Ear Punch | female | 2 | 123478649 | 125123408 | 0.32 |
| Ear Hole Area Six Weeks After Ear Punch | female | 4 | 101882585 | 106748034 | 0.34 |
| Ear Hole Area Six Weeks After Ear Punch | female | 4 | 139583632 | 140528737 | 0.27 |
| Ear Hole Area Six Weeks After Ear Punch | female | 7 | 88739954 | 91417412 | 1 |
| Ear Hole Area Six Weeks After Ear Punch | female | 11 | 6153573 | 8402096 | 0.54 |
| Ear Hole Area Six Weeks After Ear Punch | female | 15 | 49175417 | 54944833 | 0.3 |
| Ear Hole Area Six Weeks After Ear Punch | female | 18 | 15166567 | 16097068 | 0.54 |
| Ear Hole Area Six Weeks After Ear Punch | male | 1 | 92906418 | 95933814 | 0.27 |
| Ear Hole Area Six Weeks After Ear Punch | male | 1 | 160302534 | 160621550 | 0.33 |
| Ear Hole Area Six Weeks After Ear Punch | male | 1 | 172771717 | 173155436 | 0.43 |
| Ear Hole Area Six Weeks After Ear Punch | male | 2 | 164200011 | 165221683 | 0.43 |
| Ear Hole Area Six Weeks After Ear Punch | male | 3 | 141912370 | 144143601 | 0.46 |
| Ear Hole Area Six Weeks After Ear Punch | male | 4 | 50880971 | 56035903 | 0.43 |
| Ear Hole Area Six Weeks After Ear Punch | male | 7 | 89127286 | 90186386 | 1 |
| Ear Hole Area Six Weeks After Ear Punch | male | 18 | 15408156 | 22319899 | 0.66 |
| Elevated Plus Maze Closed Arm Distance | female | 6 | 71326285 | 76742046 | 0.33 |
| Elevated Plus Maze Closed Arm Distance | female | 11 | 37583470 | 39697632 | 0.6 |
| Elevated Plus Maze Closed Arm Distance | female | 11 | 41281706 | 46029422 | 0.3 |
| Elevated Plus Maze Closed Arm Distance | female | 14 | 71920012 | 74227679 | 0.25 |
| Elevated Plus Maze Closed Arm Distance | male | 1 | 18595589 | 20623798 | 0.31 |
| Elevated Plus Maze Closed Arm Distance | male | 9 | 41870531 | 44657615 | 0.33 |
| Elevated Plus Maze Closed Arm Distance | male | 11 | 92322471 | 96137410 | 0.61 |
| Elevated Plus Maze Open Arm Distance | female | 2 | 3242596 | 9751188 | 0.27 |
| Elevated Plus Maze Open Arm Distance | female | 2 | 99342120 | 101424004 | 0.3 |
| Elevated Plus Maze Open Arm Distance | female | 5 | 57923864 | 59926204 | 0.36 |
| Elevated Plus Maze Open Arm Distance | female | 8 | 73838970 | 76584375 | 0.9 |
| Elevated Plus Maze Open Arm Distance | female | 12 | 24809520 | 27578230 | 0.41 |
| Elevated Plus Maze Open Arm Distance | male | 3 | 49558738 | 53064239 | 0.34 |
| Elevated Plus Maze Open Arm Distance | male | 5 | 45049616 | 46967291 | 0.6 |
| Elevated Plus Maze Open Arm Distance | male | 9 | 107385155 | 110408954 | 0.75 |
| Elevated Plus Maze Open Arm Distance | male | X | 148556976 | 153742601 | 0.36 |
| Startle Response | female | 1 | 171780175 | 172980758 | 0.26 |
| Startle Response | female | 11 | 107458150 | 108977608 | 0.76 |
| Startle Response | female | 14 | 74925298 | 77890117 | 0.45 |
| Startle Response | female | 15 | 90718436 | 92378459 | 1 |
| Startle Response | male | 2 | 170652373 | 172529097 | 0.31 |
| Startle Response | male | 4 | 87106864 | 88541977 | 0.48 |
| Startle Response | male | 4 | 90379743 | 98986670 | 0.62 |
| Startle Response | male | 6 | 108599580 | 112015741 | 0.25 |
| Startle Response | male | 7 | 36124756 | 37554905 | 0.27 |
| **Startle Response** | **male** | **11** | **116531783** | **121529538** | **1** |
| Startle Response | male | 15 | 90718436 | 92992022 | 0.99 |
| Startle Response | male | 17 | 62731881 | 63149098 | 0.3 |
| Startle Response | male | X | 61868716 | 65868119 | 0.38 |
| Glucose Levels After 0 Minutes | female | 2 | 160636812 | 161566006 | 0.5 |
| Glucose Levels After 0 Minutes | female | 3 | 25789217 | 30655403 | 0.84 |
| Glucose Levels After 0 Minutes | female | 7 | 53518649 | 56717200 | 0.4 |
| Glucose Levels After 0 Minutes | female | 11 | 22652379 | 24119207 | 0.54 |
| Glucose Levels After 0 Minutes | male | 3 | 19163210 | 21265573 | 0.46 |
| Glucose Levels After 0 Minutes | male | 6 | 62802636 | 63772977 | 0.27 |
| Glucose Levels After 0 Minutes | male | 11 | 5429786 | 5855027 | 0.35 |
| Glucose Levels After 0 Minutes | male | 14 | 47816920 | 49506830 | 0.33 |
| Glucose Levels After 75 Minutes | female | 4 | 127433785 | 129249377 | 0.69 |
| Glucose Levels After 75 Minutes | female | X | 103283648 | 104607924 | 0.46 |
| Area Under Curve of Glucose Levels | female | 4 | 127074444 | 129607739 | 0.86 |
| Area Under Curve of Glucose Levels | female | 5 | 13823258 | 18071808 | 0.39 |
| Area Under Curve of Glucose Levels | female | 8 | 126492366 | 127638433 | 0.4 |
| Area Under Curve of Glucose Levels | male | 7 | 62522641 | 65367018 | 0.82 |
| Area Under Curve of Glucose Levels | male | 12 | 14719531 | 17739797 | 0.87 |
| Basophils | male | 17 | 3484561 | 7341224 | 0.25 |
| Basophils | male | 19 | 41357732 | 46090064 | 0.72 |
| Hematocrit | female | 1 | 163955713 | 169192742 | 0.45 |
| Hematocrit | female | 9 | 63514332 | 65312870 | 0.35 |
| Hematocrit | female | 11 | 89105622 | 89761781 | 0.4 |
| Hematocrit | male | 2 | 166801701 | 168792085 | 0.26 |
| Hematocrit | male | 3 | 73675097 | 78034163 | 0.25 |
| Hematocrit | male | 13 | 34306545 | 41426435 | 0.31 |
| Hemoglobin | female | 1 | 165540465 | 166007751 | 0.28 |
| Hemoglobin | female | 1 | 167838459 | 169192742 | 0.45 |
| Hemoglobin | female | 5 | 139536184 | 141093822 | 0.27 |
| Hemoglobin | female | 11 | 88977887 | 89938811 | 0.49 |
| Hemoglobin | male | 17 | 80478769 | 81351500 | 0.51 |
| Hemoglobin | male | 18 | 56618586 | 58717432 | 0.4 |
| Lymphocytes | female | 2 | 135137887 | 135592454 | 0.46 |
| Lymphocytes | female | 18 | 28318348 | 29763500 | 0.3 |
| Lymphocytes | female | X | 9430856 | 10276927 | 0.34 |
| Lymphocytes | male | 3 | 30655403 | 33964549 | 0.28 |
| Lymphocytes | male | 5 | 107112442 | 115597784 | 0.26 |
| Lymphocytes | male | 14 | 9425077 | 11494028 | 0.64 |
| Lymphocytes | male | 15 | 64914027 | 65833577 | 0.25 |
| Lymphocytes | male | 15 | 74802356 | 75435371 | 0.37 |
| Lymphocytes | male | 16 | 39493346 | 43610777 | 0.86 |
| Mean Cellular Hemoglobin | female | 1 | 128625610 | 141417567 | 0.81 |
| Mean Cellular Hemoglobin | female | 5 | 99880059 | 102108827 | 0.44 |
| Mean Cellular Hemoglobin | female | 6 | 83505914 | 84840195 | 0.38 |
| Mean Cellular Hemoglobin | female | 8 | 75173575 | 80766342 | 0.48 |
| Mean Cellular Hemoglobin | female | 8 | 83200393 | 84568855 | 0.59 |
| Mean Cellular Hemoglobin | female | 9 | 107917976 | 111255564 | 0.97 |
| Mean Cellular Hemoglobin | female | 10 | 82455510 | 83110573 | 0.32 |
| Mean Cellular Hemoglobin | female | 14 | 69379696 | 72653712 | 0.64 |
| Mean Cellular Hemoglobin | female | 15 | 78989686 | 87306911 | 0.52 |
| Mean Cellular Hemoglobin | male | 6 | 83292891 | 84746715 | 0.51 |
| Mean Cellular Hemoglobin | male | 7 | 113660134 | 117906779 | 0.85 |
| Mean Cellular Hemoglobin | male | 8 | 125471492 | 128414758 | 0.5 |
| Mean Cellular Hemoglobin | male | 11 | 5535137 | 6167305 | 0.63 |
| Mean Cellular Hemoglobin | male | 11 | 99302132 | 104023311 | 0.45 |
| Mean Cellular Hemoglobin | male | 14 | 70786590 | 71177943 | 0.95 |
| Mean Cellular Volume | female | 1 | 128523738 | 135115263 | 0.99 |
| Mean Cellular Volume | female | 3 | 128047779 | 128958345 | 0.3 |
| Mean Cellular Volume | female | 8 | 83200393 | 84644018 | 0.38 |
| Mean Cellular Volume | female | 9 | 108991958 | 113824027 | 1 |
| Mean Cellular Volume | female | 11 | 5535137 | 6153573 | 0.34 |
| Mean Cellular Volume | female | 14 | 69379696 | 72989668 | 0.89 |
| Mean Cellular Volume | male | 1 | 128464682 | 134993149 | 0.97 |
| Mean Cellular Volume | male | 2 | 72933792 | 75021839 | 0.4 |
| Mean Cellular Volume | male | 11 | 17204963 | 18566602 | 0.37 |
| Mean Cellular Volume | male | 13 | 94170898 | 96470882 | 0.41 |
| Mean Cellular Volume | male | 14 | 51202517 | 51637210 | 0.79 |
| Mean Cellular Volume | male | 14 | 68750445 | 71177273 | 0.26 |
| Mean Cellular Volume | male | 14 | 86187292 | 87234247 | 0.94 |
| Mean Cellular Volume | male | 19 | 9972150 | 12068136 | 0.7 |
| Mean platelet volume | female | 1 | 172771717 | 176723784 | 1 |
| Mean platelet volume | female | 3 | 13643132 | 14887794 | 0.47 |
| Mean platelet volume | female | 4 | 63558958 | 66377177 | 0.57 |
| Mean platelet volume | female | 5 | 16300800 | 17827135 | 0.41 |
| Mean platelet volume | female | 9 | 107473108 | 110701061 | 0.39 |
| Mean platelet volume | female | 14 | 45858301 | 47467088 | 0.69 |
| Mean platelet volume | female | X | 138602978 | 139242562 | 0.29 |
| Mean platelet volume | male | 1 | 140096429 | 142430268 | 0.43 |
| Mean platelet volume | male | 1 | 172771717 | 173155436 | 0.98 |
| Mean platelet volume | male | 2 | 10478353 | 11989969 | 0.62 |
| Mean platelet volume | male | 2 | 115832014 | 120302483 | 0.26 |
| Mean platelet volume | male | 3 | 149089096 | 152287221 | 0.42 |
| Mean platelet volume | male | 4 | 134294713 | 138420378 | 0.34 |
| Mean platelet volume | male | 16 | 12237574 | 12887956 | 0.26 |
| Neutrophil count | female | 1 | 186146131 | 188452609 | 0.37 |
| Neutrophil count | female | 4 | 135488839 | 136775221 | 0.26 |
| Neutrophil count | female | 18 | 29519995 | 34074222 | 0.54 |
| Neutrophil count | male | 1 | 80719179 | 83524951 | 0.55 |
| Neutrophil count | male | 1 | 130357492 | 132515586 | 0.25 |
| Plateletcrit | female | 3 | 50235065 | 55060180 | 0.29 |
| Plateletcrit | female | 9 | 34266328 | 34646073 | 0.33 |
| Plateletcrit | female | 12 | 112426248 | 114385312 | 0.41 |
| Plateletcrit | female | 13 | 93113198 | 96283561 | 0.25 |
| Plateletcrit | male | 7 | 47652751 | 51559741 | 0.32 |
| Plateletcrit | male | 11 | 103369194 | 104434921 | 0.72 |
| Plateletcrit | male | 19 | 55093306 | 58525051 | 0.48 |
| Platelets | female | 1 | 173176208 | 174420652 | 0.59 |
| Platelets | female | 9 | 26263345 | 29112775 | 0.3 |
| Platelets | female | 18 | 56763024 | 57397815 | 0.46 |
| Platelets | female | 18 | 62145797 | 64232516 | 0.25 |
| Platelets | male | 4 | 81082042 | 87302984 | 0.36 |
| Platelets | male | 10 | 11485905 | 14123091 | 0.48 |
| Platelets | male | 11 | 96942297 | 100250921 | 0.69 |
| Platelets | male | 15 | 3927809 | 6270679 | 0.31 |
| Red Blood Cell Count | female | 1 | 165927223 | 169192742 | 0.77 |
| Red Blood Cell Count | male | 11 | 81061772 | 83225930 | 0.3 |
| Red Blood Cell Count | male | 15 | 86437448 | 88522520 | 0.36 |
| Red Blood Cell Count | male | 17 | 80770639 | 81351500 | 0.76 |
| White Blood Cell Count | female | 2 | 135137887 | 135592454 | 0.26 |
| White Blood Cell Count | female | 4 | 135849702 | 136594254 | 0.26 |
| White Blood Cell Count | female | 11 | 68849550 | 70371199 | 0.53 |
| White Blood Cell Count | female | 11 | 91481888 | 95706399 | 0.27 |
| White Blood Cell Count | female | 13 | 83876493 | 88799450 | 0.49 |
| White Blood Cell Count | female | 19 | 40657408 | 46961471 | 0.27 |
| White Blood Cell Count | male | 1 | 144498343 | 148052886 | 0.29 |
| White Blood Cell Count | male | 7 | 87470824 | 93725019 | 0.36 |
| White Blood Cell Count | male | 8 | 14480079 | 16233475 | 0.64 |
| White Blood Cell Count | male | 14 | 8923855 | 11494028 | 0.51 |
| White Blood Cell Count | male | 15 | 74988859 | 75435371 | 0.29 |
| White Blood Cell Count | male | 16 | 38069109 | 43943528 | 0.68 |
| CD4+:CD8+ Ratio | female | 1 | 53399835 | 55043651 | 0.38 |
| CD4+:CD8+ Ratio | female | 1 | 57243479 | 59435875 | 0.27 |
| CD4+:CD8+ Ratio | female | 5 | 13998275 | 16842022 | 0.29 |
| CD4+:CD8+ Ratio | female | 5 | 112765005 | 115597784 | 0.48 |
| CD4+:CD8+ Ratio | female | 6 | 68817518 | 74677758 | 0.5 |
| CD4+:CD8+ Ratio | female | 12 | 89933366 | 95234080 | 0.54 |
| CD4+:CD8+ Ratio | female | 17 | 34167248 | 40707958 | 1 |
| CD4+:CD8+ Ratio | female | 17 | 41425377 | 41918576 | 0.48 |
| CD4+:CD8+ Ratio | female | 17 | 45461862 | 48002496 | 0.36 |
| CD4+:CD8+ Ratio | male | 3 | 115356189 | 119424749 | 0.25 |
| CD4+:CD8+ Ratio | male | 6 | 71323583 | 73776008 | 0.3 |
| CD4+:CD8+ Ratio | male | 6 | 103069280 | 107015161 | 0.26 |
| CD4+:CD8+ Ratio | male | 9 | 37007330 | 47075366 | 0.49 |
| CD4+:CD8+ Ratio | male | 11 | 5855027 | 6774647 | 0.36 |
| CD4+:CD8+ Ratio | male | 12 | 26818120 | 27258227 | 0.3 |
| CD4+:CD8+ Ratio | male | 17 | 33077989 | 37570713 | 1 |
| CD4+ Intensity | female | 3 | 112653405 | 115646074 | 0.36 |
| CD4+ Intensity | female | 4 | 129249377 | 134644420 | 0.53 |
| CD4+ Intensity | female | 6 | 77510828 | 82693471 | 0.59 |
| CD4+ Intensity | female | 10 | 47262385 | 48405428 | 0.42 |
| CD4+ Intensity | female | 12 | 10538257 | 10663632 | 0.59 |
| CD4+ Intensity | female | 14 | 38075998 | 40378447 | 0.78 |
| CD4+ Intensity | male | 1 | 172771717 | 176081703 | 0.75 |
| CD4+ Intensity | male | 5 | 104357881 | 108813581 | 0.32 |
| CD4+ Intensity | male | 6 | 125312051 | 125667401 | 0.4 |
| CD4+ Intensity | male | 9 | 29542937 | 31419441 | 0.31 |
| CD4+ Intensity | male | 11 | 98624873 | 100625636 | 0.79 |
| CD4+ Intensity | male | 16 | 31242726 | 32194765 | 0.42 |
| CD4+ Intensity | male | 16 | 91372479 | 91679059 | 0.26 |
| CD8+ Intensity | female | 9 | 29147731 | 31723273 | 0.29 |
| CD8+ Intensity | female | 17 | 41761386 | 43560014 | 0.32 |
| CD8+ Intensity | female | 17 | 69398741 | 69740044 | 0.32 |
| CD8+ Intensity | male | 3 | 90414133 | 91607832 | 0.26 |
| CD8+ Intensity | male | 4 | 53915257 | 56035903 | 0.46 |
| CD8+ Intensity | male | 8 | 111470906 | 112837135 | 0.3 |
| CD8+ Intensity | male | 12 | 43449827 | 46245477 | 0.72 |
| B220+ Cells | female | 1 | 45566104 | 48200927 | 0.34 |
| B220+ Cells | female | 1 | 120379270 | 122503972 | 0.31 |
| B220+ Cells | female | 1 | 130357492 | 130928323 | 0.47 |
| B220+ Cells | female | 2 | 50372238 | 53042542 | 0.26 |
| B220+ Cells | female | 6 | 134571704 | 136698174 | 0.43 |
| B220+ Cells | female | 12 | 25988592 | 28665483 | 0.91 |
| B220+ Cells | female | 12 | 71468454 | 74144220 | 0.37 |
| **B220+ Cells** | **male** | **1** | **93932364** | **97723675** | **0.88** |
| B220+ Cells | male | 2 | 68682089 | 69180806 | 0.33 |
| B220+ Cells | male | 2 | 118137997 | 128959365 | 0.89 |
| B220+ Cells | male | 4 | 64960920 | 70061092 | 0.41 |
| B220+ Cells | male | 5 | 84265009 | 85890122 | 0.38 |
| B220+ Cells | male | 9 | 39934634 | 42066624 | 0.39 |
| B220+ Cells | male | 11 | 32646121 | 33063196 | 0.4 |
| B220+ Cells | male | 16 | 94664355 | 96723377 | 0.76 |
| B220+ Cells | male | 18 | 64820731 | 68284961 | 0.25 |
| B220+ Cells | male | 19 | 60875841 | 61266404 | 0.74 |
| CD3+ Count | female | 1 | 44668125 | 48361345 | 0.41 |
| CD3+ Count | female | 2 | 149749907 | 152530292 | 0.39 |
| CD3+ Count | female | 3 | 113786274 | 121501316 | 0.55 |
| CD3+ Count | female | 4 | 130004333 | 131083434 | 0.31 |
| CD3+ Count | female | 6 | 134571704 | 137411248 | 0.85 |
| CD3+ Count | female | 9 | 54508169 | 55292556 | 0.32 |
| CD3+ Count | female | 12 | 25779635 | 28665483 | 0.54 |
| CD3+ Count | female | 12 | 103766611 | 104892589 | 0.31 |
| CD3+ Count | male | 1 | 95905962 | 100503409 | 0.26 |
| CD3+ Count | male | 2 | 122449872 | 128959389 | 0.68 |
| CD3+ Count | male | 7 | 118398316 | 127927413 | 0.52 |
| CD3+ Count | male | 9 | 32645815 | 38061700 | 0.36 |
| CD4+ Count | female | 1 | 123887380 | 125550610 | 0.3 |
| CD4+ Count | female | 3 | 68871235 | 69819671 | 0.29 |
| CD4+ Count | female | 3 | 87619539 | 93138626 | 0.26 |
| CD4+ Count | female | 12 | 25779635 | 28665483 | 0.26 |
| CD4+ Count | female | 15 | 87533260 | 87908042 | 0.58 |
| CD4+ Count | male | 2 | 99507457 | 101541181 | 0.31 |
| CD4+ Count | male | 6 | 116847401 | 119361386 | 0.35 |
| CD4+ Count | male | 8 | 73838970 | 75068826 | 0.54 |
| CD4+ Count | male | 9 | 37007330 | 37748928 | 0.99 |
| CD4+ Count | male | 11 | 32860837 | 37583470 | 0.29 |
| CD4+ Count | male | 19 | 52034977 | 52276420 | 0.29 |
| CD4+ Cells in CD3+ Cells | female | 3 | 75061591 | 77716516 | 0.38 |
| CD4+ Cells in CD3+ Cells | female | 5 | 112765005 | 114910345 | 0.58 |
| CD4+ Cells in CD3+ Cells | female | 6 | 68817518 | 74638679 | 0.29 |
| CD4+ Cells in CD3+ Cells | female | 12 | 89933366 | 95234080 | 0.33 |
| CD4+ Cells in CD3+ Cells | female | 17 | 33155241 | 41918576 | 1 |
| CD4+ Cells in CD3+ Cells | female | 17 | 45706483 | 48002496 | 0.43 |
| CD4+ Cells in CD3+ Cells | male | 1 | 169192742 | 175676780 | 0.43 |
| CD4+ Cells in CD3+ Cells | male | 3 | 25789217 | 30845875 | 0.33 |
| CD4+ Cells in CD3+ Cells | male | 6 | 4763401 | 9787412 | 0.32 |
| CD4+ Cells in CD3+ Cells | male | 6 | 71323583 | 73776008 | 0.25 |
| CD4+ Cells in CD3+ Cells | male | 9 | 37007330 | 47075366 | 0.7 |
| CD4+ Cells in CD3+ Cells | male | 11 | 5855027 | 6513859 | 0.53 |
| CD4+ Cells in CD3+ Cells | male | 17 | 33077989 | 37658709 | 1 |
| CD8+ Count | female | 1 | 130485574 | 130928323 | 0.25 |
| CD8+ Count | female | 1 | 177945546 | 178339586 | 0.44 |
| CD8+ Count | female | 6 | 68817518 | 75834607 | 0.54 |
| CD8+ Count | female | 6 | 134399785 | 135272017 | 0.7 |
| CD8+ Count | female | 8 | 29288832 | 31235115 | 0.91 |
| CD8+ Count | female | 12 | 25779635 | 29344130 | 0.43 |
| CD8+ Count | female | 12 | 91589224 | 94983892 | 0.34 |
| CD8+ Count | female | 17 | 32390322 | 34517928 | 1 |
| CD8+ Count | female | 17 | 40707958 | 42803695 | 1 |
| CD8+ Count | male | 1 | 169017790 | 169890101 | 0.48 |
| CD8+ Count | male | 1 | 172343863 | 175676780 | 0.44 |
| CD8+ Count | male | 6 | 103069280 | 104683826 | 0.4 |
| CD8+ Count | male | 11 | 4131025 | 7285878 | 0.89 |
| CD8+ Count | male | 11 | 95489315 | 96817154 | 0.38 |
| CD8+ Count | male | 17 | 34329744 | 40807075 | 1 |
| CD8+ Count | male | 17 | 43961485 | 47054970 | 0.51 |
| CD8+ Cells in CD3+ Cells | female | 1 | 53399835 | 55611951 | 0.44 |
| CD8+ Cells in CD3+ Cells | female | 1 | 57243479 | 59435875 | 0.36 |
| CD8+ Cells in CD3+ Cells | female | 5 | 14824301 | 16842022 | 0.3 |
| CD8+ Cells in CD3+ Cells | female | 5 | 112765005 | 116005573 | 0.38 |
| CD8+ Cells in CD3+ Cells | female | 6 | 68817518 | 75834607 | 0.53 |
| CD8+ Cells in CD3+ Cells | female | 12 | 89933366 | 95234080 | 0.52 |
| CD8+ Cells in CD3+ Cells | female | 17 | 33765847 | 38311620 | 1 |
| CD8+ Cells in CD3+ Cells | female | 17 | 41347509 | 42580589 | 0.59 |
| CD8+ Cells in CD3+ Cells | female | 17 | 45823630 | 48002496 | 0.27 |
| CD8+ Cells in CD3+ Cells | male | 6 | 103069280 | 105083552 | 0.46 |
| CD8+ Cells in CD3+ Cells | male | 11 | 5855027 | 6774647 | 0.6 |
| CD8+ Cells in CD3+ Cells | male | 12 | 109759370 | 113032555 | 0.31 |
| CD8+ Cells in CD3+ Cells | male | 17 | 33077989 | 37570713 | 1 |
| Area Under Curve of Insulin Levels | female | 12 | 113741390 | 115578692 | 0.37 |
| Area Under Curve of Insulin Levels | male | 2 | 161565938 | 162677301 | 0.37 |
| Area Under Curve of Insulin Levels | male | 4 | 104187278 | 108752970 | 0.41 |
| Area Under Curve of Insulin Levels | male | 10 | 59795986 | 60567967 | 0.33 |
| Area Under Curve of Insulin Levels | male | 13 | 118295239 | 120179440 | 0.42 |
| Body Mass Index | female | 12 | 87124120 | 88386384 | 0.52 |
| Body Mass Index | male | 1 | 84300299 | 90246244 | 0.32 |
| Body Mass Index | male | 2 | 115248529 | 120302445 | 0.29 |
| Body Mass Index | male | 2 | 126929073 | 128300235 | 0.5 |
| Body Mass Index | male | 4 | 3630716 | 5606295 | 0.37 |
| Body Mass Index | male | 6 | 90102227 | 91311931 | 0.34 |
| Body Mass Index | male | 11 | 98342741 | 101112093 | 0.57 |
| Body Mass Index | male | 14 | 21661895 | 24010972 | 0.31 |
| Body Length | male | 1 | 84300299 | 92906418 | 0.52 |
| Fecal Boli Count in Open Field Test | female | 10 | 73132593 | 74981509 | 0.25 |
| Fecal Boli Count in Open Field Test | female | 11 | 3454099 | 7106175 | 0.6 |
| Fecal Boli Count in Open Field Test | female | 18 | 65605243 | 69905227 | 0.31 |
| Fecal Boli Count in Open Field Test | male | 3 | 18466041 | 19163210 | 0.69 |
| Activity in New Home Cage | female | 1 | 3397373 | 6645944 | 0.55 |
| Activity in New Home Cage | female | 1 | 45566104 | 47260916 | 0.34 |
| Activity in New Home Cage | female | 2 | 7832634 | 10142362 | 0.67 |
| Activity in New Home Cage | female | 4 | 80950026 | 87055009 | 0.25 |
| Activity in New Home Cage | female | 11 | 21655867 | 22652379 | 0.27 |
| Activity in New Home Cage | female | 12 | 45826130 | 54936906 | 0.31 |
| Activity in New Home Cage | female | 14 | 38908201 | 39961649 | 0.39 |
| Activity in New Home Cage | female | 14 | 119355200 | 120110781 | 0.25 |
| Activity in New Home Cage | male | 7 | 47173175 | 47652751 | 0.39 |
| Activity in New Home Cage | male | 11 | 20218491 | 24572649 | 0.6 |
| Activity in New Home Cage | male | 15 | 30285053 | 34371808 | 0.54 |
| Respiratory Rate | female | 1 | 63624905 | 66461385 | 0.34 |
| Respiratory Rate | female | 4 | 148493933 | 149135934 | 0.73 |
| Respiratory Rate | female | 6 | 109902083 | 110763328 | 0.28 |
| Respiratory Rate | male | 1 | 74335917 | 76887544 | 0.37 |
| Respiratory Rate | male | 1 | 133649413 | 137179674 | 0.31 |
| Respiratory Rate | male | 1 | 190268338 | 191159138 | 0.4 |
| Respiratory Rate | male | 2 | 57512164 | 58748595 | 0.39 |
| Respiratory Rate | male | 9 | 107473108 | 108700691 | 0.32 |
| Respiratory Rate | male | 15 | 3927809 | 5426959 | 0.25 |
| Tidal Volume | female | 11 | 92924770 | 96166802 | 0.66 |
| Tidal Volume | female | 12 | 34644286 | 37051142 | 0.25 |
| Tidal Volume | male | 8 | 95465648 | 97213397 | 0.63 |
| Tidal Volume | male | 15 | 89413198 | 89873413 | 0.37 |
| Tidal Volume | male | 16 | 32471847 | 35110554 | 0.35 |
