# Supplementary material for: Genetic Interactions with Sex Make a Relatively Small Contribution to the Heritability of Complex Traits in Mice
Source: PLoS One. 2014 May 8;9(5):e96450. doi: 10.1371/journal.pone.0096450 (PMC4014490; doi:10.1371/journal.pone.0096450)
Supplement: Table S4 — Heritability estimates across phenotypes. (DOCX) [file pone.0096450.s005.docx]

| Phenotype | *h^2^* | ± s.d. |
| --- | --- | --- |
| Adrenal Gland Weight | 0.227 | 0.031 |
| Serum Albumin | 0.170 | 0.028 |
| Serum Alkaline Phosphatase | 0.484 | 0.031 |
| Serum Alanine Transaminase | 0.198 | 0.031 |
| Serum Aspartate Aminotransferase | 0.168 | 0.031 |
| Serum Calcium | 0.280 | 0.033 |
| Serum Chloride | 0.278 | 0.033 |
| Serum High-Density Lipoprotein | 0.440 | 0.033 |
| Serum Low-Density Lipoprotein | 0.285 | 0.033 |
| Serum Phosphorous | 0.182 | 0.034 |
| Serum Sodium | 0.248 | 0.032 |
| Serum Total Protein | 0.115 | 0.028 |
| Serum Triglycerides | 0.243 | 0.036 |
| Serum Urea | 0.204 | 0.030 |
| Freeze Time to Fear-Associated Context | 0.291 | 0.040 |
| Freeze Time to Fear-Associated Cue | 0.216 | 0.025 |
| Ear Hole Area Six Weeks After Ear Punch | 0.412 | 0.037 |
| Body Weight | 0.388 | 0.033 |
| Elevated Plus Maze Closed Arm Distance | 0.255 | 0.032 |
| Elevated Plus Maze Open Arm Distance | 0.252 | 0.032 |
| Startle Response | 0.340 | 0.032 |
| Glucose Levels After 0 Minutes | 0.161 | 0.035 |
| Glucose Levels After 75 Minutes | 0.206 | 0.029 |
| Area Under Curve of Glucose Levels | 0.191 | 0.032 |
| Basophils | 0.049 | 0.031 |
| Hematocrit | 0.136 | 0.020 |
| Hemoglobin | 0.160 | 0.030 |
| Lymphocytes | 0.228 | 0.031 |
| Mean Cellular Hemoglobin | 0.584 | 0.034 |
| Mean Cellular Volume | 0.496 | 0.031 |
| Monocyte count | 0.106 | 0.034 |
| Mean platelet volume | 0.257 | 0.029 |
| Neutrophil count | 0.216 | 0.035 |
| Plateletcrit | 0.157 | 0.034 |
| Platelets | 0.204 | 0.032 |
| Red Blood Cell Count | 0.148 | 0.035 |
| White Blood Cell Count | 0.228 | 0.032 |
| CD4+:CD8+ Ratio | 0.505 | 0.033 |
| CD4+ Intensity | 0.463 | 0.035 |
| CD8+ Intensity | 0.334 | 0.037 |
| B220+ Cells | 0.460 | 0.038 |
| CD3+ Count | 0.439 | 0.036 |
| CD4+ Count | 0.418 | 0.037 |
| CD4+ Cells in CD3+ Cells | 0.476 | 0.038 |
| CD8+ Count | 0.550 | 0.036 |
| CD8+ Cells in CD3+ Cells | 0.509 | 0.033 |
| Area Under Curve of Insulin Levels | 0.176 | 0.035 |
| Body Mass Index | 0.134 | 0.031 |
| Body Length | 0.169 | 0.028 |
| Fecal Boli Count in Open Field Test | 0.137 | 0.029 |
| Respiratory Rate | 0.239 | 0.028 |
| Tidal Volume | 0.182 | 0.032 |
